# Polyphenols from Citrus Tacle^®^ Extract Endowed with HMGCR Inhibitory Activity: An Antihypercholesterolemia Natural Remedy

**DOI:** 10.3390/molecules26185718

**Published:** 2021-09-21

**Authors:** Fedora Grande, Maria Antonietta Occhiuzzi, Maria Rosaria Perri, Giuseppina Ioele, Bruno Rizzuti, Giancarlo Statti, Antonio Garofalo

**Affiliations:** 1 Department of Pharmacy, Health and Nutritional Sciences, University of Calabria, Ampl. Polifunzionale, Via P. Bucci, 87036 Rende, Italy; mariaantonietta.occhiuzzi@unical.it (M.A.O.); mariarosaria.perri@unical.it (M.R.P.); giuseppina.ioele@unical.it (G.I.); giancarlo.statti@unical.it (G.S.); antonio.garofalo@unical.it (A.G.); 2 CNR-NANOTEC, SS Rende (CS), Department of Physics, University of Calabria, Via P. Bucci, 87036 Rende, Italy; bruno.rizzuti@cnr.it; 3 Institute of Biocomputation and Physics of Complex Systems (BIFI), Joint Units IQFR-CSIC-BIFI, and GBsC-CSIC-BIFI, University of Zaragoza, 50018 Zaragoza, Spain

**Keywords:** flavanones, anti-enzymatic assays, molecular docking

## Abstract

Tacle^®^ is a citrus fruit obtained from the crossbreeding of Clementine and Tarocco cultivars. This fruit retains a promising nutraceutical potential most likely due to a high content in polyphenols, among which the main constituents are the two glycosides naringin and hesperidin. Herein, we evaluated, through an in vitro assay, the capability of Tacle extracts to inhibit the hydroxymethylglutaryl-CoA reductase enzyme, which plays a key role in cholesterol biosynthesis. The results obtained spurred us to investigate whether the anti-enzymatic activity observed may be due to a direct interaction of aglycones naringenin and hesperetin with the enzyme catalytic site. Molecular docking simulations indicated that these two compounds are able to anchor to the protein with binding modes and affinities similar to those found for statins, which represent mainstream medications against hypercholesterolemia. The overall results showed an interesting nutraceutical potential of Tacle, suggesting that its extract could be used for dietary supplementation in the treatment of moderate hypercholesterolemia.

## 1. Introduction

Hypercholesterolemia is a disorder characterized by the presence of high levels of cholesterol in the blood. As a consequence, people with hypercholesterolemia show a higher risk of cardiovascular diseases, even leading to an early heart attack. Healthy lifestyle behaviors can help reduce the risks, although remarkable high concentrations of hematic cholesterol require treatments including medications [1].

The first-line remedies against hypercholesterolemia are represented by statins, either synthetic or contained in natural extracts such as the one obtained from red yeast rice made by fermenting white rice with various strains of the mold *Monascus purpureus* [2,3]. These drugs have been shown to be capable of inhibiting hydroxymethylglutaryl-CoA reductase (HMGCR), the enzyme that plays a central role in the production of cholesterol in the liver. Nevertheless, drugs with a different mode of action are also available and can be used either alone or in combination. For example, ezetimibe, a β-lactam based inhibitor of an aminopeptidase involved in cholesterol intestinal absorption, is often formulated in combination with a statin, while bile acid sequestrants are considered as second-line remedies [4].

Very recently, bempedoic acid, a prodrug with inhibitory activity on adenosine triphosphate citrate lyase, another hepatic enzyme involved in cholesterol biosynthesis, has been introduced into clinical practice [5]. Hyperuricemia, pain in the limbs, and anemia are, however, among its most common side effects, limiting its prescription. The use of a first-in-class small interfering RNA, inclisiran [6], or monoclonal antibodies targeting PCSK9 (proprotein convertase subtilisin/kexin type 9) such as alirocumab and evolocumab is reserved exclusively to severe cases resistant to statins. In fact, inhibitors of this enzyme have been shown to increase the removal of low-density lipoproteins (LDL) from extracellular fluid, and consequently also from blood. In spite of the impressive results coming from the application of their subcutaneous long-lasting formulations, the use of such new therapeutics is limited by very high costs and possible cognitive adverse effects [7].

As a conclusion, statins remain first-line drugs generally useful in medium/severe hypercholesterolemia, although the incidence of common unwanted effects such as muscle problems, an increased risk of diabetes, and liver damage, somewhat limits their use.

An interesting alternative to synthetic drugs is represented by natural antihypercholesterolemic remedies. The majority of them are based on the use of monacolin K (lovastatin), a statin used either purified or as a component of standardized red rice powder, often associated with agents endowed with an antioxidant complementary mechanism. Among them, berberine, an alkaloid present in plants of the genus *Berberis*, has gained a certain popularity, although some risks are connected with its use. In fact, unpredictable interactions with prescription drugs and a lack of conclusive reports on its efficacy do not allow for a general clinical application for this substance. The mode of action should in this case reside in an upregulation of the LDL receptor [8]. Q10 coenzyme, polycosanol, astaxanthin, lipoic acid, and folic acid are other agents often co-formulated with monacolin K in dietary supplements and nutraceuticals.

Another approach to lower hematic cholesterol is based on the use of supplements rich in plant sterols and stanols [9], which would interfere with cholesterol intestinal absorption. The regular assumption of food containing cholesterol-normalizing active principles could represent an alternative approach, especially in the case of mild/medium alterations of blood cholesterol content. Several preparations obtained from various parts of plants belonging to the *Citrus* genus have been recognized as effective in mildly lowering hematic cholesterol. This activity was attributed to diverse compounds such as phenol derivatives, polyphenols, and sterols that are present in juices and purees of these fruits [10].

In light of these findings, the potential of Tacle peel and pulp extract was investigated in order to ascertain a hypocholesterolemic action due to its high polyphenol content. Tacle is a hybrid fruit obtained from the crossbreeding of Tarocco and Clementina oranges [11] that is largely farmed in the lowlands of northern Calabria, a southern Italian region. The extract of this fruit is rich in polyphenol glycosides, mainly naringin and hesperidin, which have already been identified as anticholesterol agents [12]. The direct assumption of Tacle extract or, as an alternative, sufficient quantities of the fruit should guarantee a high efficacy. In a previous study, we assessed the total polyphenol and flavonoid content of Tacle extract as well as their phytochemical composition [13]. Moreover, a noteworthy inhibitory activity against lipase and amylase enzymes was found for the whole extract.

Due to our continuous interest toward the utilization of Tacle as a dietary supplement, in the present work, the inhibitory activity of the extract and the two glycosides against HMGCR was investigated by in vitro studies. Furthermore, the presence of the sugar moiety in glycosides only reflects on pharmacokinetic properties, but is not required during the interaction with the target protein [14]. In fact, glycosides are demonstrated to undergo hydrolysis by microflora β-glucosidase in the colon and this is assumed to be the rate-limiting step for the aglycone absorption. Afterward, aglycones are metabolized into conjugated metabolites, which are eliminated by renal excretion within 24 h [15,16]. In light of these findings, the investigation was extended to naringenin and hesperetin, the aglycones corresponding to naringin and hesperidin, respectively. Moreover, several studies have suggested that these flavonoids do not show significant toxicological effects, allowing for their classification as low-risk nutraceuticals [17,18].

The capability of the aglycones to bind the enzyme active site was also investigated by docking studies.

## 2. Results and Discussion

### 2.1. In Vitro Studies

Following a standard procedure in collecting and manipulating plant samples, the extraction yield as well as the quantification of the total phenolic content in the Tacle extract were determined. A yield of 8.72% and an amount of 6.15 ± 0.10 and a 0.05 ± 0.004 mg of polyphenols and flavonoids, respectively, were recorded for each gram of fresh fruit material. Hesperidin and naringin content of the extract were quantified in 310.5 ± 8.4 and 188.7 ± 5.6 ppm, respectively, while the content of hesperetin and naringenin resulted in 86.3 ± 6.4 and 212.0 ± 7.8 ppm, respectively.

The extract and each pure flavonoid were successively tested in a specific HMGCR inhibition in vitro assay.

As reported in Table 1, the inhibition potency of the whole extract, at the highest tested concentration (500 µg/mL), resulted in being comparable to that obtained for the two pure aglycones. In contrast, the glycosides were less active. At lower concentrations, the Tacle extract was observed to be significantly more active than any single glycoside and aglycone. As an example, at a concentration of 300 µg/mL, the extract was twice as active as any other compound (about 30% inhibitory activity, compared to 12–14% for each single compound, with the exception of naringin, which did not show any appreciable activity at this concentration). A residual activity of the Tacle extract was observed at concentrations even lower than 300 µg/mL, whereas all single compounds were inactive. Only at concentrations as low as 50 µg/mL did the extract not show any measurable inhibitory activity. Pravastatin was used as the positive control (yielding a percentage of inhibition of 79.0 ± 5.2 at 42.45 µg/mL).

In terms of IC_50_, the Tacle extract showed an inhibitory activity against HMGCR of 452.6 ± 6.1 µg/mL, higher than that obtained for each single aglycone (IC_50_ value of 472.7 ± 6.1 and 485.4 ± 8.7 µg/mL for naringenin and hesperetin, respectively). The lack of activity for naringin and hesperidin did not allow us to calculate their IC_50_ value.

These overall results showed that the active components in the extract had a synergistic action, as the IC_50_ for the entire extract was lower than the IC_50_ of each single compound, analogously to what is reported in previous studies on similar compounds [19,20].

Taken together, our results and an already proven safety for these compounds would confirm their good potential as nutraceutical agents.

### 2.2. Molecular Docking

The ability of interaction of the ligands with the enzyme was investigated by molecular docking using six selected crystallographic structures of HMGCR co-crystallized with statins, withdrawn from the Protein Data Bank (PDB). HMGCR is a monomeric protein anchored in the membrane of endoplasmic reticulum of hepatocytes. The main human isoform comprises 888 amino acids: a membrane bound N-terminal domain (residues 1–339), a linker region (residues 340–459), and a long and conserved cytoplasmic catalytic C-terminus domain (residues 460–888) [21].

**Figure 1:**
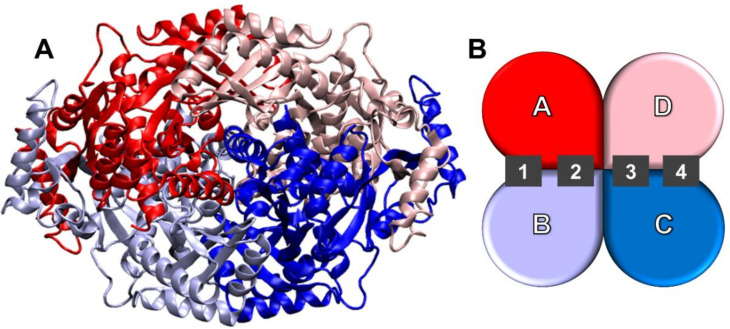
(**A**) Visualization of the tetrameric assembly of the catalytic domains of HMGCR; (**B**) Schematic representation of the arrangement of the chains (A, B, C, and D) and location of the active sites (1, 2, 3, and 4).

The catalytic portions of human HMGCR form a tightly associated tetramer (chains A, B, C, and D; Figure 1). The tetrameric assembly forms four binding sites: two of them are located at the interface between chain A and B, and the other two are positioned at the interface between chain C and D, in a symmetrical fashion. Each of the four sites can bind a (3S)-hydroxy-3-methylglutaryl-CoA (HMG-CoA) unit to be converted into mevalonic acid, the precursor of cholesterol. Statins bind to the same sites by acting as potent competitive reversible inhibitors. Due to their steric hindrance, statins have been demonstrated to be capable of precluding HMGCoA access to the catalytic pocket by distorting the entire site conformation, thereby blocking the de novo cholesterol biosynthesis [22].

Statins have been shown to be able to accommodate into each of the four active sites of HMGCR interacting with seven key amino acid residues (Glu559, Arg590, Ser684, Asp690, Lys692, Lys735, Asn755) by ionic and dipole interactions [23]. In a first step of our simulation procedure, re-docking experiments have been performed in order to calculate the binding energy value for all the crystallographic statins into each binding site of the associated tetramer (Table 2, column 3).

Subsequently, naringenin and hesperetin have been docked into each of the four active sites of the six crystallographic structures, in order to verify whether the hypocholesterolemic activity of the Tacle extract could be attributed to a direct enzyme interaction with these compounds. The overall results showed that naringenin and hesperetin interact with the protein with binding energy values ranging from −7.3 to −8.3 kcal/mol, therefore comparable to those obtained for statins (Table 2, columns 4,5).

**Figure 2:**
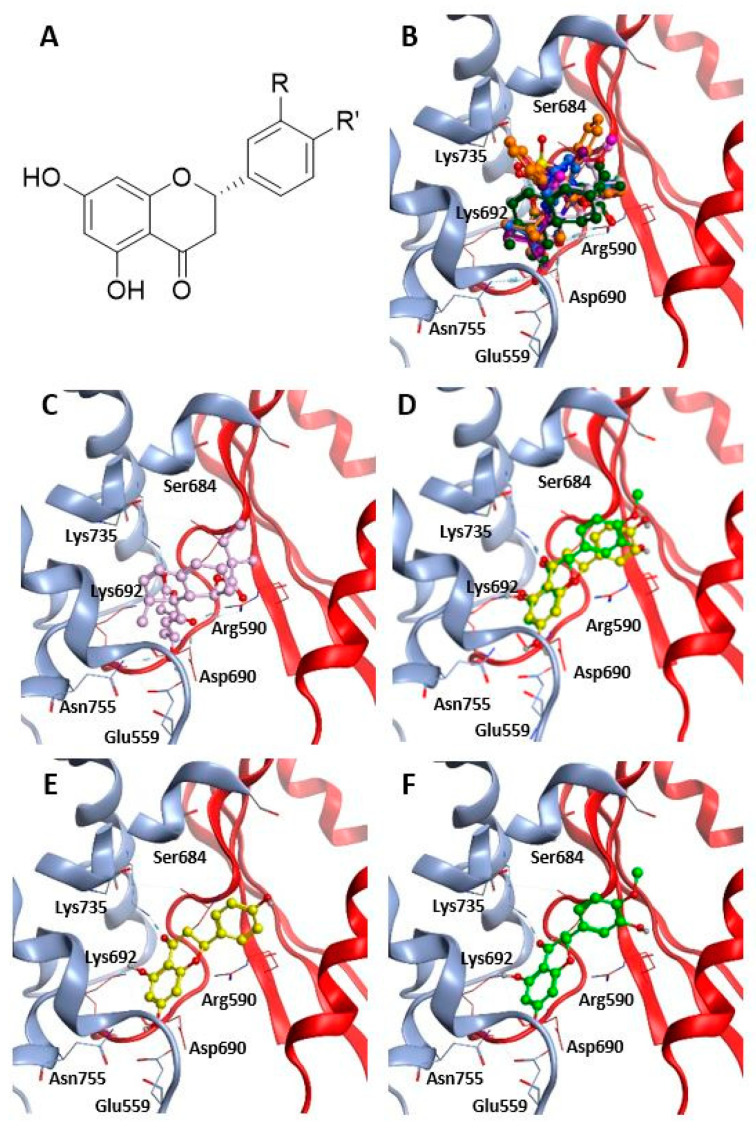
(**A**) Chemical structure of naringenin (R = H, R’ = OH) and hesperetin (R = OH, R’ = OMe); (**B**–**F**) Ligand-binding pocket of the active site of HMGCR; ribbons representing protein structural element are shown. The key residues are also indicated. (**B**) Superimposed binding modes of all the six crystallographic statins: mevastatin (pink), cerivastatin (magenta), simvastatin (dark green), rosuvastatin (purple), fluvastatin (blue), atorvastatin (orange); (**C**) binding mode of mevastatin; (**D**) superimposed binding modes of the two flavonoid ligands: naringenin (yellow) and hesperetin (light green); specific binding mode of naringenin (**E**), hesperetin (**F**).

Based on the most favorable affinity value and similarity in the orientation within the catalytic site, the docking poses were selected for the two ligands and the results are summarized in Figure 2.

Overall, naringenin and hesperetin share a similar orientation within the active site, interacting mainly with Glu559, Ser684, Lys735, Asn755 (Table 3), all residues that are also involved into statin-HMGCR interactions. This observation further supports the possibility that both naringenin and hesperetin could have a direct effect on HMGCR.

## 3. Materials and Methods

### 3.1. Chemicals

Folin–Ciocolteu reagent, aluminum chloride, sodium carbonate, HMG-CoA Reductase Assay Kit [4], naringenin, naringin, hesperitin, and hesperidin were purchased from Sigma-Aldrich S.p.A. (Milan, Italy). Acetonitrile and water were HPLC, LC-MS grade. All other solvents were of reagent grade. All solvents were purchased from VWR International (Milan, Italy).

### 3.2. Plant Material and Extraction Procedure

Tacle is a registered trademark that indicates a triploid hybrid obtained by the crossbreed of Tarocco (*Citrus sinensis* L. Osbeck) and Monreal Clementina (*Citrus clementina* Hort. ex. Tanaka) oranges. Fruit samples were collected in the lowlands of Francavilla Marittima, within the region Calabria, Italy, in December 2020. Tacle samples were collected and manipulated in the shortest time possible. Peel and pulp of fresh fruit were washed, cut into small pieces, and extracted through maceration in methanol (plant material:solvent, 1:10 g/mL, 48 h × 3 times, at room temperature). The extract obtained was filtered and brought to dryness on a rotary evaporator.

### 3.3. Total Polyphenol and Flavonoid Content Determination

#### 3.3.1. Antioxidant Power of Polyphenols

Samples were dissolved in acetone/methanol/water/acetic acid, 40:40:20:0.1 (final extract concentration 2 mg/mL). The resulting mixture was incubated in a water bath at 60 °C, and Folin–Ciocolteu reagent (1 mL) and Na_2_CO_3_ 7.5% *w*/*v* solution (1 mL) were added. Absorbance was measured at 726 nm after leaving the sample for 2 h at room temperature [24]. The result is expressed as mg of chlorogenic acid per g of fresh weight material.

#### 3.3.2. Total Flavonoid Content

AlCl_3_ (2%, 1 mL) was added to the sample (final concentration 2 mg/mL, 80% EtOH). Absorbance was measured at 430 nm after 15 min in the dark [25]. The result was expressed as mg of quercetin per g of fresh weight material [13].

#### 3.3.3. Flavanones Content

The determination of hesperidin, naringin, hesperetin, and naringenin was accomplished by HPLC following the method reported in 13.

### 3.4. HMGCR Inhibition Assay

The HMGCR inhibitory activity of the tested sample was determined spectrophotometrically by using the HMGCR Assay Kit purchased from Sigma Aldrich (Milan, Italy). The experimental procedure was modified from the original Sigma-Aldrich protocol: assay buffer 1×, NADPH, HMG-CoA, HMGCR, and 1 µL of sample at concentrations ranging from 50 to 500 µg/mL were mixed in a 96-well plate. Absorbance was measured by a microplate reader (Synergy H1 Hybrid Reader, Biotek, Winooski, VT, USA) at 340 nm after 10 min at 37 °C, in order to evaluate NADPH oxidation. Pravastatin was used as the positive control. Percentage of inhibition was calculated by using the following formula:

Inhibition (%) = [(As_1_ − As_2_)/Ac] × 100 (1)

where As_1_ is the absorbance of the sample in presence of the enzyme only; As_2_ is the absorbance of the sample in presence of both the enzyme and the substrate; and Ac is the absorbance of the control.

### 3.5. Statistical Analysis

Experiments were performed in triplicate and data were expressed as mean ± S.D. Non-linear regression analyses were performed through Graph-Pad Prism Software (San Diego, CA, USA). Statistical differences between treated groups and the control were tested by one-way analyses of variance (ANOVA), followed by Dunnett’s multiple comparison test (SigmaPlot Software, SanRafael, CA, USA).

### 3.6. Molecular Docking

Molecular docking was performed on the crystallographic structure of the tetramer of the catalytic portions of human HMGCR (in complex with statins), corresponding to PDB entry 1HW8 (co-crystallized with mevastatin), 1HW9 (simvastatin), 1HWI (fluvastatin), 1HWJ (cerivastatin), 1HWK (atorvastatin), and 1HWL (rosuvastatin) [26]. Molecular structures of naringenin and hesperetin (both in their naturally occurring *S* enantiomeric form) were built by using the modeling software Avogadro [27]. Docking calculations were performed by using AutoDock Vina 1.1.2 [28]. Preliminary conversion of the structures from the PDB format was carried out by the graphical interface AutoDock Tools 1.5.6 [29]. During the conversion, polar hydrogens were added to the crystallographic enzyme structures, whereas apolar hydrogens of the ligands were merged to the carbon atom they are attached to. Full flexibility was guaranteed for the ligands, resulting in four active torsions for naringenin and five for hesperetin. A single simulation run was carried out in each case at very high exhaustiveness, 16 times larger than the default value [30]. The binding modes of the ligands were analyzed through visual inspection, while interaction energies and distances were quantified by using Molecular Operating Environment (MOE) 2018.01 (Chemical Computing Group ULC, Montreal, MTL, Canada).

## 4. Conclusions

The extract of the citrus hybrid Tacle has been studied as a potential anticholesterol dietary supplement. The nutraceutical properties of this fruit are due to its remarkable polyphenol content. The extract, particularly rich in naringin and hesperidin, was shown to be effective against HMGCR in a specific in vitro assay. In our experiments, the whole extract proved to exert HMGCR inhibitory action more potent than the two single glycosides, even when considered in their aglycone form. Moreover, docking studies showed that naringenin and hesperetin are able to interact with key residues of the enzyme active site in a manner similar to statins and with a comparable binding energy. The overall results suggest that, after an accurate standardization in its polyphenol content, the Tacle extract could be further investigated to be included in specific diets for the management of hypercholesterolemia.

## Figures and Tables

**Table 1:** Percentage of HMGCR inhibition.

Concentrations	% Inhibition				
	Tacle Extract	Naringin	Hesperidin	Naringenin	Hesperetin
500 µg/mL	55.5 ± 1.1 *	17.3 ± 1.1 *	43.2 ± 1.6 *	53.8 ± 1.5 *	55.7 ± 3.0 *
400 µg/mL	46.7 ± 0.9 *	8.3 ± 1.1 **	30.9 ± 2.7 *	36.9 ± 1.8 *	23.2 ± 2.2 *
300 µg/mL	29.6 ± 2.1 *	-	12.2 ± 0.6 *	13.2 ± 1.5 *	13.9 ± 1.4 *
200 µg/mL	21.4 ± 0.9 *	-	-	-	-
100 µg/mL	12.3 ± 1.7 *	-	-	-	-

Data were expressed as mean ± S.D. (n = 3). * *p* < 0.001, ** *p* < 0.05 compared to the control (Dunnett’s multiple comparison test).

**Table 2:** Binding energy values obtained in the re-docking of statins in the crystallographic complexes and in the docking of naringenin and hesperetin in each receptor binding site.

**Protein Structure**		Binding Energy * **(kcal/mol)**		
**PDB Code**	**Crystallographic Ligands**	**Ligand Re-Docking**	**Naringenin**	**Hesperetin**
1HW8	mevastatin	−6.53 ± 0.22	−7.65 ± 0.58	−7.57 ± 0.15
1HW9	simvastatin	−6.59 ± 0.27	−7.70 ± 0.05	−7.60 ± 0.14
1HWI	fluvastatin	−7.88 ± 0.38	−7.67 ± 0.15	−7.55 ± 0.13
1HWJ	cerivastatin	−7.21 ± 0.12	−7.80 ± 0.24	−7.45 ± 0.17
1HWK	atorvastatin	−9.11 ± 0.04	−7.62 ± 0.09	−7.42 ± 0.05
1HWL	rosuvastatin	−7.57 ± 0.04	−7.77 ± 0.05	−7.47 ± 0.09

* Average and standard deviation over the four HMGCR binging sites.

**Table 3:** Key residues of HMGCR active sites interacting with the ligands.

PDB Entry	Site	Naringenin	Hesperetin
1HW8	1	Lys735 B	Ser684 A, Lys735 B
	2	Lys735 A	Glu559 A, Lys735 A
	3	Lys735 D	Glu559 D, Glu665 C, Glu665 C, Lys735 D, Lys691 C
	4	Lys735 C	Lys735 C
1HW9	1	Lys735 B, Asn755 B	Ser684 A, Lys735 B
	2	Glu559 A, Lys735 A, Ser684 B, Lys691 B	Glu559 A, Lys735 A, Ser684 B, Lys691 B
	3	Glu665 C, Lys735 D	Glu665 C, Ser684 C, Lys735 D
	4	Glu665 D, Lys735 C	Lys735 C, Ser684 D, Asn755 C
1HWI	1	Lys735 B, Asn755b	Glu559 B, Ser684 A, Lys735 B
	2	Ala751 A, Glu665 B, Lys735 A	Glu665 B, Lys735 A
	3	Ala751 D, Ser684 C, Lys735 D	Ala751 D, Ser684 C, Lys735 D
	4	Lys735 C, Ser661 D	Lys735 C
1HWJ	1	Lys735 B	Lys735 B
	2	Lys735 A	Lys735 A, Ser684 B
	3	Lys735 D	Lys735 D
	4	Glu665 D, Lys735 C	Glu559 C, Lys735 C, Lys691 D
1HWK	1	Glu665 A, Lys735 B	Ser684 A, Lys735 B
	2	Glu559 A, Glu665 B, Lys735 A, Lys691 B	Glu665 B, Lys735 A, Ser684 B
	3	Ala751 D, Lys735 D, Asn755 D	Lys735 D
	4	Glu665 D, Lys735 C	Glu559 C, Lys735 C, Lys691 D
1HWL	1	Ser684 A, Lys735 B	Glu559 B, Ser684 A, Lys735 B
	2	Lys735 A, Ser684 B	Lys735 A, Ser684 B
	3	Ser684 C, Lys735 D	Lys735 D
	4	Lys735 C, Ser684 D	Glu559 C, Glu665 D, Lys735 C, Ser684 D

## Data Availability

Not applicable.

## Sample Availability

Samples of the compounds are not available from the authors.
